# Differing Causes of Lactic Acidosis and Deep Breathing in Cerebral Malaria and Severe Malarial Anemia May Explain Differences in Acidosis-Related Mortality

**DOI:** 10.1371/journal.pone.0163728

**Published:** 2016-09-29

**Authors:** Nathan R. Brand, Robert O. Opoka, Karen E. S. Hamre, Chandy C. John

**Affiliations:** 1 Columbia University College of Physicians and Surgeons, School of Medicine, New York, New York, United States of America; 2 Makerere University, Department of Pediatrics, Kampala, Uganda; 3 University of Minnesota, Department of Pediatrics, Minneapolis, Minnesota, United States of America; 4 Indiana University, Department of Pediatrics, Indianapolis, Indiana, United States of America; Université Pierre et Marie Curie, FRANCE

## Abstract

Lactic acidosis (LA) is a marker for mortality in severe malaria, but the mechanisms that lead to LA in the different types of severe malaria and the extent to which LA-associated mortality differs by type of severe malaria are not well described. We assessed the frequency of LA in children admitted to Mulago Hospital, Kampala, Uganda with cerebral malaria (CM, n = 193) or severe malarial anemia (SMA, n = 216). LA was compared to mortality and measures of parasite biomass and sequestration (*P*. *falciparum* histidine-rich protein-2 (PfHRP2) concentration, platelet count), and to a measure of systemic tissue oxygen delivery (hemoglobin level). LA was more frequent in children with SMA than CM (SMA, 47.7%, CM, 34.2%, *P* = 0.006), but mortality was higher in children with CM (13.0%) than SMA (0.5%, *P*<0.0001). In CM, LA was associated with increased PfHRP2 concentration and decreased platelet count but was not associated with hemoglobin level. In contrast, in SMA, LA was associated with a decreased hemoglobin level, but was not associated with PfHRP2 concentration or platelet count. LA was related to mortality only in CM. In multivariable regression analysis of the effect PfHRP2 and hemoglobin levels on LA and DB, only *Pf*HRP2 level increased risk of LA and DB in CM, while in SMA, elevated hemoglobin strongly decreased risk of LA and DB, and *Pf*HRP2 level modestly increased risk of LA. The study findings suggest that LA in CM is due primarily to parasite sequestration, which currently has no effective adjunctive therapy, while LA in SMA is due primarily to anemia, which is rapidly corrected with blood transfusion. Differing etiologies of LA in CM and SMA may explain why LA is associated with mortality in CM but not SMA.

## Introduction

The World Health Organization estimates that in 2015 there were 214 million new cases of malaria and 438,000 deaths [1]. 90% of these deaths occurred in sub-Saharan Africa, 77% were children under the age of 5, and 66% occurred within 24 hours of hospital admission[1, 2]. Lactic acidosis (LA) has been useful as a marker of disease severity in other infectious disease processes such as sepsis [3], and has been associated with mortality in multiple studies of severe malaria [4–7]. Since measurement of LA requires diagnostic equipment not available in many health centers in sub-Saharan Africa, researchers have assessed whether a clinical proxy would provide similar information. Respiratory distress (RD), a summary diagnosis comprised of nasal flaring, chest in-drawing, intercostal recessions, and deep breathing (DB) correlated strongly with LA [8], and studies by English et al investigating each component of RD separately found that DB was the most sensitive and specific indicator of severe metabolic acidosis in children with severe malaria [9]. The observation was confirmed by the Severe Malaria in African Children (SMAC) network, a network comprising more than 14,000 children in 3 countries, which found DB to be one of three clinical signs used in the Lambaréné organ dysfunction score to predict mortality in hospitalized patients with malaria [10].

Studies to date on the associations between LA, DB or RD and mortality have assessed children with severe malaria as a single clinical entity and have not assessed risks in the different types of severe malaria independently. Severe malarial anemia (SMA) is among the most common forms of severe malaria, with an estimated 1.5 to 5 million children affected annually [11]. Reported mortality rates in SMA vary widely from 1.7% [12] to 9% [13], and are consistently lower than the mortality rates for cerebral malaria (CM), which is less common but more often fatal, with an average 18.6% mortality [14]. Diverse etiologies can lead to acidosis in children suffering from different forms of severe malaria, so the reversal of LA requires knowledge of what is causing the LA. Potential contributors to LA in children include local tissue parasite sequestration, with resulting localized ischemia, which may occur in any form of severe malaria, but is likely most pronounced in CM, and systemic impairment of oxygen delivery due to low hemoglobin levels, which is most common in SMA.

To resolve the questions of how LA, RD and DB relate to each other, how they differ in two of the most common forms of severe malaria, how mortality with each factor differs, and which processes contribute to LA, RD and DB in each form of severe malaria, we assessed LA, RD and DB, along with markers of parasite biomass and sequestration and markers of systemic perfusion and oxygen delivery in a cohort of Ugandan children with CM or SMA.

## Methods

### Study Population

The study was performed at Mulago Hospital, Kampala, Uganda. The primary goal of the study was to assess the presence of neurocognitive impairment in children with CM or SMA, and pathogenesis of disease in these two forms of malaria. Children with CM, or SMA were enrolled if they were between 18 months and 12 years of age. CM was defined as: 1) coma (Blantyre Coma Score [BCS]≤2 or Glasgow Coma Score [GSC]≤8); 2) *Plasmodium falciparum* on blood smear; 3) no other known cause of coma (e.g., meningitis, a prolonged postictal state or hypoglycemia-associated coma reversed by glucose infusion). SMA was defined as presence of *Plasmodium falciparum* on blood smear in children with hemoglobin level ≤ 5 g/dL. Hemoglobin was measured by photometry (HemoControl; EKF Diagnostics). Exclusion criteria for all children included: 1) known chronic illness requiring medical care; 2) known developmental delay; or 3) prior history of coma, head trauma, hospitalization for malnutrition, or cerebral palsy. Additional exclusion criteria for children with SMA included 1) impaired consciousness to any degree on physical exam (BCS<5); 2) other clinical evidence of CNS disease; or 3) >1 seizure prior to admission.

Children with CM or SMA were managed according to the Ugandan Ministry of Health treatment guidelines current at the time of the study. These included initial intravenous quinine treatment followed by oral quinine for severe malaria while admitted, artemisinin combination therapy for outpatient follow-up therapy, and blood transfusion for all children with a hemoglobin ≤5 g/dL (which included all children with SMA, including children with concurrent CM and SMA).

### Clinical and laboratory assessment

Respiratory distress (RD) was defined as the presence of acidotic breathing, nasal flaring, intercostal recessions, or chest indrawing, and deep breathing (DB) was defined solely by the presence of deep acidotic breathing. Plasma *P*. *falciparum* histidine-rich protein-2 (*Pf*HRP2) levels were tested to assess parasite biomass and sequestration [15], and platelet counts measured to assess for platelet contribution to sequestration [16]. To assess systemic perfusion and oxygen delivery, hemoglobin levels and oxygen saturation were assessed.

On admission, venous blood was collected for microscopy and lactate measurement. Blood smears for microscopy were Giemsa stained and read independently by two readers, with discordant results resolved by a third reader. Lactate was measured using a point of care lactate analyzer (Accutrend lactate meter, Roche Diagnostics, Mannheim, Germany). Lactic acidosis was defined as a blood lactate level > 5.0 mmol/L. Plasma *Pf*HRP2 levels were quantified using the commercially available Malaria Ag CELISA kit (Cellabs, Brookvale, Australia). The enzyme linked immunosorbent assays were performed according to the manufacturer’s protocol with optical density (OD) determined at 450 and 620 nm. Plasma was diluted 1:2400 in the kit-provided 1X PBS/Tween buffer (PBST). Samples with results above or below the range of the standard curve were retested at a dilution of 1:24,000 or 1:200, respectively. To assess intra-assay repeatability, 10% of samples (from a mix of study groups) from each assay plate were replicated on subsequent plates. The coefficient of variation for these samples was 19.6%. Total, circulating and sequestered parasite biomass were calculated using a formula adapted from Dondorp et al. [17] and Cunnington et al [18], in which total parasite biomass is 7.3* PfHRP2*(1- (hematocrit*0.01))*weight*(10^7), circulating biomass is parasite density*(10^6)*(0.08*weight), and sequestered parasite biomass is total biomass–circulating biomass.

### Data Analysis

Data was entered into a FileMaker Pro 11 database (FileMaker Inc, Santa Clara, CA), and exported to and analyzed in Stata SE 12 (Stata Corporation, College Station, TX). Categorical variables were analyzed using the chi-squared test, and continuous variables were assessed using the Wilcoxon rank sum test. Logistic regression was used to explore the predictive power of LA, RD or DB on mortality. *Pf*HRP2 levels and platelet count were not normally distributed and so were natural log transformed for regression analysis.

### Ethical approval

Written informed consent was obtained from parents or guardians of study participants. The Institutional Review Boards for human studies at Makerere University School of Medicine and the University of Minnesota granted ethical approval for the study.

## Results

### Demographic, clinical and laboratory findings in children with CM compared to children with CM and SMA

**Fig 1:**
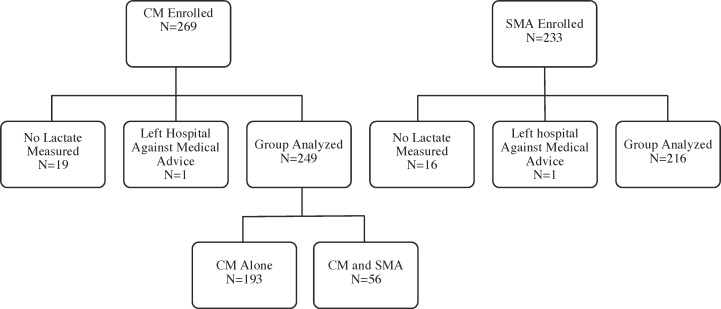
Study enrollment and testing for lactic acidosis.

A total of 269 children with CM and 233 children with SMA were enrolled in the study. 1 child with CM and 1 with SMA left the hospital against medical advice, and 19 children with CM and 16 children with SMA did not have lactate level measured. Analysis was done on the remaining 249 children with CM and 216 children with SMA (Fig 1). Children with CM or SMA who had LA measured did not differ from those who did not have LA measured in terms of mortality (CM, 12.4% vs. 15.8%, P = 0.7; SMA, 0.5% vs 0%, P = 1.0) or frequency of deep breathing (CM, 8.8% vs. 10.5%, P = 0.7. SMA, 7.4% vs. 0%, P = 0.6).

Of the 249 children with CM, 56 had concurrent SMA. Children with CM and concurrent SMA differed from children with CM alone in several aspects: they were significantly younger, and had more frequent respiratory distress and deep breathing, lower hemoglobin levels, higher *Pf*HRP2 levels, and lower peripheral *P*. *falciparum* parasite density than children with CM alone (S1 Table). Despite these differences, mortality did not differ between children with CM alone (13.0%) and children with CM and SMA (10.7%). Because we wanted to explore how risk factors differed specifically in CM as compared to SMA, primary analysis was conducted comparing children with CM and no SMA (n = 193) to children with SMA alone (none of whom had CM). The subsequent analyses refer to these groups, but S1–S5 Tables describe the same findings as Tables 1–5 for children with both CM and SMA compared to children with CM alone.

**Table 1:** Demographic, clinical and laboratory findings children with cerebral malaria alone (CM) or severe malarial anemia alone (SMA) on admission.

Characteristic or finding	CM	SMA	*P*^*a*^
	N = 193	N = 216	
Age in years, mean (SD)	4.2 (2.0)	3.4 (1.7)	<0.0001
Sex, N female (%)	75 (38.9)	84 (38.9)	0.9
Mortality, N (%)	25 (13.0)	1 (0.5)	<0.0001
Respiratory distress, N (%)	47 (24.4)	65 (30.1)	0.2
Deep breathing, N (%)	12 (6.2)	16 (7.4)	0.6
Lactic acidosis^*b*^, N (%)	66 (34.2)	103 (47.7)	0.006
Blood lactate, mmol/L, median (IQR)	3.8 (2.3, 6.5)	4.8 (2.9, 8.1)	0.001
Hemoglobin, g/dL, mean (SD)	7.8 (1.9)	3.7 (0.9)	<0.0001
O_2_ Saturation, median (IQR)	97 (94, 99)	98 (95, 99)^*c*^	0.5
O2 Saturation, <92%, N (%)	19 (9.8)	15 (6.9)^*c*^	0.3
Platelet count, 10^9^/L, median (IQR)	56 (35, 103)^*c*^	154 (96, 234)^*c*^	<0.0001
PfHRP2, 10^3^ng/mL, median (IQR)	2,561 (881, 5,148)	881 (367, 2,581)^*c*^	<0.0001
Peripheral blood *P falciparum* density, parasites/μL, median (IQR)	53,120 (13,980, 340,770)^*c*^	35,740 (10,100, 156,040)^*c*^	0.005

^*a*^ P-value for continuous variables compared by Students’ t-test if normally distributed and Wilcoxon rank-sum if skewed distribution, and for categorical variables by χ^2^ or Fisher’s exact test where appropriate

^*b*^ Lactic acidosis defined as blood lactate > 5.0 mmol/liter

^*c*^ N differs from total N and is noted in S6 Table

**Table 2:** Parasite biomass.

	CM	SMA	*P*^*a*^
	N = 166	N = 179	
Total parasite biomass x 10^8^, median (IQR)	17,427 (5,747, 37,523)	7,572 (2,371, 18,214)	<0.0001
Circulating parasite biomass x 10^8^, median (IQR)	643 (170, 3,814)	410 (124, 1,676)	0.004
Sequestered parasite biomass x 10^8^, median (IQR)	13,717 (4,628, 32,886)	5,963 (1,546, 16,177)	<0.0001
	CM Deep Breathing	CM No Deep Breathing	
	N = 9	N = 157	
Total parasite biomass x 10^8^, median (IQR)	44,831 (19,859, 202,395)	16,610 (5,314, 34,895)	0.01
Circulating parasite biomass x 10^8^, median (IQR)	3,534 (868, 7,583)	588 (169, 3,574)	0.07
Sequestered parasite biomass x 10^8^, median (IQR)	32,525 (16,325, 197,712)	13,273 (4,535, 30,851)	0.02
	CM lactic acidosis	CM No lactic acidosis	
	N = 57	N = 109	
Total parasite biomass x 10^8^, median (IQR)	21,460 (11,688, 42,333)	15,019 (3,792, 32,887)	0.03
Circulating parasite biomass x 10^8^, median (IQR)	1,092 (327, 4,721)	488 (117, 3,271)	0.05
Sequestered parasite biomass x 10^8^, median (IQR)	16,325 (8,405, 39,820)	12,362 (2,487, 30, 851)	0.05
	SMA Deep Breathing	SMA No Deep Breathing	
	N = 9	N = 170	
Total parasite biomass x 10^8^, median (IQR)	6,910 (5,238, 32,009)	7,677 (2,251, 17,364)	0.3
Circulating parasite biomass x 10^8^, median (IQR)	869 (378, 2,769)	397 (119, 1,597)	0.1
Sequestered parasite biomass x 10^8^, median (IQR)	6,249 (3,322, 28,502)	5,892 (1,546, 16,030)	0.6
	SMA lactic acidosis	SMA No lactic acidosis	
	N = 75	N = 104	
Total parasite biomass x 10^8^, median (IQR)	8,620 (2,904, 22,886)	6,260 (2,215, 14,081)	0.05
Circulating parasite biomass x 10^8^, median (IQR)	400 (99, 1,731)	427 (133, 1,636)	1.0
Sequestered parasite biomass x 10^8^, median (IQR)	8,001 (2,056, 22,334)	5,109 (1,471, 12,515)	0.07
	CM Died	CM Survived	
	N = 22	N = 144	
Total parasite biomass x 10^8^, median (IQR)	33,493 (13,481, 52,668)	16,351 (5,208, 32,983)	0.008
Circulating parasite biomass x 10^8^, median (IQR)	1,069 (242, 3,240)	578 (170, 4,111)	0.8
Sequestered parasite biomass x 10^8^, median (IQR)	31,957 (12,290, 51,668)	13,011 (3,197, 29,789)	0.006

^*a*^ P-value for Wilcoxon rank-sum

**Table 3:** Levels of disease pathogenesis markers in children with cerebral malaria and severe malaria anemia with vs. without deep breathing or lactic acidosis.

Cerebral Malaria			
	Deep breathing	No deep breathing	P^*a*^
	N = 12	N = 181	
Platelet Count 10^9^/L, median (IQR)	34 (14, 43)^*b*^	59 (35, 108)^*b*^	0.008
PfHRP210^3^ng/ml, median (IQR)	7,291 (3,550, 19,175)	2,486 (812, 4,894)	0.0005
Hemoglobin, g/dL, mean (SD)	7.2 (1.5)	7.8 (2.0)	0.3
Peripheral blood *P falciparum* Density^*b*^	365,840 (47,880, 789,940)^*c*^	49,765 (13,100, 295,060)^*c*^	0.05
	Lactic acidosis	No lactic acidosis	
	N = 66	N = 127	
Platelet Count 10^9^/L, median (IQR)	42 (28, 62)^*c*^	65 (38, 130)^*c*^	0.0003
PfHRP2 10^3^ng/ml, median (IQR)	3,335 (1,680, 7,281)	2,244 (542, 4,507)	0.0005
Hemoglobin, g/dL, mean (SD)	7.5 (2.0)	7.9 (1.9)	0.2
Peripheral blood *P falciparum* Density^*b*^	114,240 (29,000, 448,360)^*c*^	42,890 (10,220, 248,210)^*c*^	0.009
Severe Malaria Anemia				
	Deep breathing	No deep breathing	P^*a*^
	N = 16	N = 200	
Platelet Count 10^9^/L, median (IQR)	175 (118, 347)	151 (95, 226)^*b*^	0.3
PfHRP210^3^ng/ml, median (IQR)	1,387 (515, 3,264)^*c*^	848 (312, 2,578)^*c*^	0.2
Hemoglobin, g/dL, mean (SD)	3.3 (0.9)	3.8 (0.9)	0.03
Peripheral blood *P falciparum* Density^*b*^	37,570 (1,506, 256,710)	35,180 (10,660, 151,760)^*c*^	0.8
	Lactic acidosis	No lactic acidosis	
	N = 103	N = 113	
Platelet Count 10^9^/L, median (IQR)	160 (96, 281)^*c*^	148 (94, 224)^*c*^	0.7
PfHRP2 10^3^ng/ml, median (IQR)	952 (312, 3,134)^*c*^	865 (371, 2,065)^*c*^	0.2
Hemoglobin, g/dL, mean (SD)	3.4 (0.9)	4.0 (0.7)	<0.0001
Peripheral blood *P falciparum* Density^*b*^	32,983 (3,110, 123,180)^*c*^	41,360 (12,380, 178,020)	0.3

^*a*^ P-value for continuous variables compared by Students’ t-test if normally distributed and Wilcoxon rank-sum if skewed distribution, and for categorical variables by χ^2^ or Fisher’s exact test where appropriate

^*b*^ Parasites/μL, median (IQR)

^*c*^ N differs from total N and is noted in S6 Table.

**Table 4:** Demographic, clinical and laboratory findings children with cerebral malaria (CM) who survived vs. died.

Characteristic or finding	Died	Survived	P^a^
	N = 25	N = 168	
Age in years, mean (SD)	3.5 (1.7)	4.3 (2.1)	0.08
Sex, N (% female)	10 (40.0)	65 (38.7)	0.9
Respiratory distress, N (%)	9 (36.0)	38 (22.6)	0.1
Deep breathing, N (%)	6 (24.0)	6 (3.6)	<0.0001
Lactic acidosis^b^, N (%)	13 (52.0)	53 (31.6)	0.04
Blood lactate, mmol/L, median (IQR)	5.1 (2.8, 7.8)	3.7 (2.2, 6.2)	0.09
Hemoglobin, g/dL, mean (SD)	8.2 (1.8)	7.7 (2.0)	0.3
O_2_ Saturation, median (IQR)	96 (93, 98)	98 (95, 99)	0.07
O2 Saturation <92%, n (%)	5 (20.0)	14 (8.3)	0.07
Platelet count, 10^9^/L, median (IQR)	41 (17, 62)^*c*^	60 (35, 110)^*c*^	0.02
PfHRP2, 10^3^ng/mL, median (IQR)	4,514 (1,598, 7,517)	2,389 (834, 4,921)	0.02
Peripheral blood *P falciparum* density^*d*^,	83,680 (21,240, 340,770)^*c*^	48,640 (12,920, 368,820)^*c*^	0.5

^*a*^ P-value for continuous variables compared by Students’ t-test if normally distributed and Wilcoxon rank-sum if skewed distribution, and for categorical variables by χ^2^ or Fisher’s exact test where appropriate

^*b*^ Lactic acidosis defined as blood lactate > 5.0 mmol/liter

^*c*^ N differs from total N and is noted in S6 Table

^*d*^ Parasites/μL, median (IQR)

**Table 5:** Mortality in children with cerebral malaria (CM) or severe malarial anemia (SMA) in the presence or absence of deep breathing and lactic acidosis.

	CM			SMA		
	N = 193			N = 216		
Finding	N with factor	% with factor	Mortality with factor^*a*^	N with factor	% with factor	Mortality with factor^*a*^
All children	193	-	25 (13.0)	216	-	1 (0.5)
DB	12	6.2	6 (50.0)	16	7.4	0
LA	66	34.2	13 (19.7)	103	47.7	0
DB and LA	12	6.2	6 (50.0)	15	6.9	0
DB without LA	0	0	0	1	0.5	0
LA without DB	54	28.0	7 (13.0)	88	40.7	0
No DB	181	93.8	19 (10.5)	200	92.6	1 (0.5)
No LA	127	65.8	12 (9.5)	113	52.3	1 (0.9)

^*a*^ N (%)

### Demographic, clinical and laboratory findings in children with CM as compared to children with SMA

More than 60% of children with CM or SMA were male, and children with CM were significantly older than children with SMA. Mortality was significantly higher in children with CM (13.0%) than in children with SMA (0.5%, *P*<0.0001, Table 1). DB occurred at similar frequencies in children with CM or SMA, but LA was more common and lactic acid levels were higher in children with SMA than CM (Table 1). Children with CM had higher PfHRP2 levels, higher peripheral *P*. *falciparum* parasite density, and lower platelet count (all factors associated with parasite biomass and sequestration) than children with SMA, while children with SMA had lower hemoglobin levels (associated with less systemic tissue oxygen delivery) than children with CM. Children with CM had higher total, circulating and sequestered parasite biomass than children with SMA (Table 2).

### Risk factors for deep breathing (DB) and lactic acidosis (LA) in children with CM as compared to children with SMA

Children with CM who had DB or LA had markers of increased parasite biomass and sequestration (higher PfHRP2, lower platelet count) compared to children with CM and no DB or LA, but the groups did not differ in hemoglobin levels (Table 3). In contrast, children with SMA and DB or LA had lower hemoglobin levels than children with SMA and no DB or LA, but the groups did not differ in markers of increased parasite biomass or sequestration (Table 3). Supporting this association, lactate levels were inversely correlated with hemoglobin level in children with SMA (Spearman’s rho = -0.3, *P*<0.0001) but not in children with CM (Spearman’s rho = -0.07, *P* = 0.3). Multivariable regression analysis of hemoglobin and log-transformed PfHRP2 showed that for children with CM, only increased PfHRP2 correlated with an increased risk of LA or DB (odds ratio (OR) [95% confidence interval (CI)], LA: *Pf*HRP2, 1.54 [1.20, 1.99], *P* = 0.001, hemoglobin, 0.97 [0.82, 1.15], *P* = 0.76; DB: *Pf*HRP2, 2,67 [1.48, 4.82], *P* = 0.001, hemoglobin, 0.97 [0.67, 1.40], *P* = 0.86). For children with SMA, elevated hemoglobin strongly decreased risk of LA, but increased PfHRP2 level was less strongly associated with an increased risk of LA (*Pf*HRP2, 1.25 [1.05, 1.48], *P* = 0.01, hemoglobin, 0.39 [0.28, 0.57], *P*<0.001), and similarly elevated hemoglobin decreased and elevated PfHRP2 increased the risk of DB, the latter just shy of statistical significance (*Pf*HRP2, 1.45 [0.98, 2.16], *P* = 0.06, hemoglobin, 0.48 [0.25, 0.90], *P* = 0.02). In children with both CM and SMA, neither hemoglobin level nor PfHRP2 was significantly associated with LA or DB (OR [95% CI], LA: *Pf*HRP2, 1.27 [0.76, 2.13], *P* = 0.36, hemoglobin, 0.57 [0.27, 1.21], *P* = 0.15; DB: *Pf*HRP2, 0.83 [0.46, 1.47], *P* = 0.52, hemoglobin, 0.92 [0.37, 2.30], *P* = 0.86)).

In children with CM, total and sequestered parasite biomass were both higher for children with DB or LA. Circulating biomass was higher in children with LA and showed a trend toward increase in children with DB (*P* = 0.07, Table 2). In children with SMA, values for total, circulating, and sequestered parasite biomass did not differ significantly in children with vs. without DB, but children with SMA and LA did have higher total parasite biomass than children without LA, and children with SMA and LA also showed a trend toward higher sequestered parasite biomass (*P* = 0.07, Table 2).

### Risk factors for mortality in children with CM

Risk factors for mortality were assessed only in children with CM. (Low mortality in children with SMA precluded analysis of risk factors). A higher proportion of children with CM who died had DB and LA than CM survivors, and children with CM who died showed markers of greater parasite biomass and sequestration (higher PfHRP2 levels, lower platelet counts) than children who survived CM (Table 4). Total and sequestered but not circulating parasite biomass were higher in children with CM who died than in those who survived (Table 2). We further assessed how DB and LA, alone or in combination, related to mortality in children with CM (Table 5). Children with DB (all of whom had LA) had the highest mortality rate (50.0%). Children with LA and no DB had a similar mortality rate to the overall cohort of children with CM (13.0%), and mortality was modestly lower in children without DB (10.2%) or without LA (9.5%). Among children with CM who received a transfusion, transfusion was not associated with a decrease in mortality (data not shown).

In a regression model including all factors associated with mortality (DB or LA, PfHRP2 level and platelet count), only DB was significantly associated with mortality (OR 4.3, 95% CI 1.1–17.1). Of note, the one child with SMA who died had neither DB nor LA (Table 3).

## Discussion

In the present study, we show that LA occurs at similar frequency in CM and SMA, but correlates with mortality only in CM. We also show that the pathophysiologic correlates of LA are different in the two forms of severe malaria: in CM, LA correlates with increased parasite biomass and decreased platelet count, both factors associated with infected red blood cell sequestration, while in SMA, DB and LA primarily correlate with lower hemoglobin level, a factor associated with impaired tissue oxygen delivery, and correlate to a lesser extent with parasite biomass. The study findings suggest that in CM, LA is associated with greater parasite sequestration, for which there is currently no effective adjunctive therapy, while in SMA, LA is primarily associated with anemia and consequent impaired tissue oxygen delivery, which are rapidly corrected with blood transfusion. The differing primary etiologies of LA in CM and SMA may explain why LA is associated with mortality in CM but not SMA, and suggest that interventions to address LA in children with severe malaria must address the underlying cause of LA, which may differ in the different forms of severe malaria.

Multiple pathophysiologic processes can lead to LA. In studies of lactic acidosis in sepsis, for example, pathways that lead to LA include factors that lead to systemic or regional hypoxia, as well as factors not associated with hypoxia, such as underlying organ disease, drugs and toxins, and inborn errors of metabolism [19]. In the present study, LA appears to be associated with processes leading to systemic hypoxia in SMA and localized hypoxia and ischemia in CM. The present study and others have shown that prompt blood transfusions can reverse the inadequate O_2_ delivery associated with LA or DB in patients with SMA and will lead to resolution of LA and low mortality rates [20, 21]. The pathophysiology of platelet adhesion and parasite sequestration, however, is not easily reversed. As a result, LA and DB in CM have been associated with a significantly higher mortality rate in multiple studies of African children [7, 22, 23]. In sepsis and other illnesses, interventions specifically addressing the elevated lactate concentration or acidosis, such as administration of dichloroacetate [24] or bicarbonate [25], have not resulted in improved survival, even though in the case of dichloroacetate, acidemia improved. Dichloroacetate decreased mortality in an animal model of severe malaria [26], but the studies in sepsis and the findings of the present study suggest that interventions that can decrease or reverse the underlying cause of LA, presumably the sequestration process in children with CM, are likely to have greater benefit than interventions like dicholoroacetate. Trials of interventions that could decrease sequestration, e.g., low anticoagulant heparin derivatives [27], are therefore urgently needed, as they have the potential to decrease mortality and morbidity in CM.

In the present study, we show that parasite biomass appears to play a role in both CM and SMA, as total and sequestered biomass were higher in children with LA than without LA in both groups. However, thrombocytopenia was associated with LA only in children with CM. Laboratory studies have shown that platelets aid in the cytoadhesion of parasitized red blood cells and could be pathologically important to cerebral malaria [16, 28]. Post mortem studies of children with CM confirm these findings, reporting a significant increase of platelets in the brain microvasculature of children with CM as compared to children without it [29], and involvement of monocytes in platelets in sequestration in children with fatal CM [30]. The association of thrombocytopenia with LA in CM but not SMA in the present study is consistent with a contribution of platelet adhesion to sequestration in the brain and other organs, and supports the idea that sequestration, involving not only sequestered parasites but platelet adhesion and likely leukocyte adhesion (a factor more difficult to measure in peripheral blood samples), leads to LA in CM. Sequestration could also play a role in the LA seen in SMA, but the strong correlation of hemoglobin level with LA in SMA and not CM, and the low mortality in SMA with blood transfusion, both suggest that the primary cause of LA in SMA is a low hemoglobin level and the resulting systemic hypoxia and not sequestration. Children with both CM and SMA did not have significant associations of either hemoglobin level or *Pf*HRP2 level with LA or DB, but the strongest association seen was of elevated hemoglobin with a decreased risk of LA (hemoglobin, OR, 95% CI, 0.57 [0.27, 1.21], *P* = 0.15), suggesting that children with both CM and SMA, LA is more strongly related to hemoglobin level than parasite biomass, i.e., that LA is driven most by the SMA component in these children.

Studies in malaria have shown a stronger relationship of base deficit than LA with mortality [31], and recent studies have shown the presence of previously unmeasured organic acids are also associated with mortality in Asian adults with severe malaria [32]. In the present study, the presence of DB but not RD was significantly associated with mortality in children with CM (*P* = <0.0001 and *P* = 0.1 respectively), confirming the observations of English et al [9] and the Severe Malaria in African Children (SMAC) network [10], that DB is the most useful respiratory sign in predicting mortality in children with malaria. DB emerged as the only independent risk factor for death when included in a multivariable regression model with *Pf*HRP2 and platelet count, suggesting that the clinical manifestation of DB may be an “end pathway” result of the sequestration reflected by high *Pf*HRP2 levels and low platelet counts.

The low mortality rate in children with SMA and LA in the present study, similar to that of a previous study of Ugandan children with SMA and LA (mortality 1.4%), demonstrates the benefit of rapid effective transfusion in SMA. In contrast, the similar mortality rates of children with CM in the present study (13.0%) and in an observational study published twenty years ago (16.8%) [33] show the need for effective adjunctive therapy in CM.

DB/metabolic acidosis alone, even in the absence CM or SMA, is an important form of severe malaria. The present study design did not include children with this form of severe malaria, but an ongoing study by our group in Uganda does include children with DB/metabolic acidosis as well as children with CM and SMA. When this study is completed, we will be able to compare the contribution of parasite biomass and hemoglobin to DB/LA and mortality in the three major forms of severe malaria: CM, SMA and DB/LA without CM or SMA. In this ongoing study, we are also assessing base deficit in addition to LA and DB, as this measure directly assesses acidosis from multiple acids in addition to LA. Our findings suggest LA may not be a good surrogate marker of mortality in studies of severe malaria that include SMA, as LA was common in SMA but did not predict mortality in this condition.

In conclusion, the present study provides evidence that LA and DB are caused by different primary pathogenic mechanisms in CM and SMA. The differences in mechanisms may explain why LA and DB are associated with mortality in CM but not SMA. The study findings suggest that in CM, LA and DB are associated with organ and tissue level sequestration, a process for which adjunctive therapies are currently unavailable, while in SMA, LA and DB are associated with anemia and consequent tissue hypoxia, which can be rapidly corrected by blood transfusion. The study findings support continued investigation of interventions to prevent or reduce parasite sequestration and platelet adhesion in cerebral malaria.

## Supporting Information

S1 Dataset (XLS)

S1 Table Demographic, clinical and laboratory findings in children with cerebral malaria alone (CM) or cerebral malaria with severe malarial anemia (CM + SMA). (DOCX)

S2 Table Parasite Biomass in children with cerebral malaria alone (CM) or cerebral malaria with severe malarial anemia (CM + SMA). (DOCX)

S3 Table Levels of disease pathogenesis markers in children with cerebral malaria (CM) and severe malaria anemia (SMA) with vs. without deep breathing or lactic acidosis. (DOCX)

S4 Table Demographic, clinical and laboratory findings in children with cerebral malaria (CM) and severe malaria anemia (SMA) who survived vs. died. (DOCX)

S5 Table Mortality in children with cerebral malaria (CM) and severe malaria anemia (SMA) in the presence or absence of deep breathing and lactic acidosis. (DOCX)

S6 Table Numbers tested for subgroup testing in prior tables. (DOCX)
