# Supplementary material for: Differing Causes of Lactic Acidosis and Deep Breathing in Cerebral Malaria and Severe Malarial Anemia May Explain Differences in Acidosis-Related Mortality
Source: PLoS One. 2016 Sep 29;11(9):e0163728. doi: 10.1371/journal.pone.0163728 (PMC5042445; doi:10.1371/journal.pone.0163728)
Supplement: S1 Table — (DOCX) [file pone.0163728.s002.docx]

**S1 Table. Demographic, clinical and laboratory findings in children with cerebral malaria alone (CM) or cerebral malaria with severe malarial anemia (CM + SMA)**

| Characteristic or finding | CM  N=193 | CM + SMA  N=56 | *P^a^* |
| --- | --- | --- | --- |
| Age in years, mean (SD) | 4.2 (2.0) | 3.3 (1.4) | 0.002 |
| Sex, N female (%) | 75 (38.9) | 23 (41.1) | 0.8 |
| Mortality, N (%) | 25 (13.0) | 6 (10.7) | 0.7 |
| Respiratory distress, N (%) | 47 (24.4) | 24 (42.9) | 0.007 |
| Deep breathing, N (%) | 12 (6.2) | 10 (17.9) | 0.007 |
| Lactic acidosis*^b^*, N (%) | 66 (34.2) | 23 (41.1) | 0.3 |
| Blood lactate, mmol/L, median (IQR) | 3.8 (2.3, 6.5) | 3.8 (2.0, 8.9) | 0.7 |
| Hemoglobin, g/dL, mean (SD) | 7.8 (1.9) | 4.1 (0.8) | <0.0001 |
| O_2_ Saturation, median (IQR) | 97 (94, 99) | 97 (94, 98) | 0.4 |
| O2 Saturation, <92%, N (%) | 19 (9.8) | 8 (14.3) | 0.3 |
| Platelet count, 10^9^/L, median (IQR) | 56 (35, 103)*^c^* | 76 (41, 126) | 0.06 |
| PfHRP2, 10^3^ng/mL, median (IQR) | 2,561 (881, 5,148) | 4,040 (1,861, 6,000) | 0.01 |
| Peripheral blood *P falciparum* density, parasites/μL, median (IQR) | 53,120 (13,980, 340,770)*^c^* | 21,750 (8,000, 161,360)*^c^* | 0.03 |

*^a^* P-value for continuous variables compared by Students’ t-test if normally distributed and Wilcoxon rank-sum if skewed distribution,

and for categorical variables by χ^2^ or Fisher’s exact test where appropriate

*^b^* Lactic acidosis defined as blood lactate > 5.0 mmol/liter

*^c^* N differs from total N and is noted in supplementary table 6
