# Supplementary material for: Differing Causes of Lactic Acidosis and Deep Breathing in Cerebral Malaria and Severe Malarial Anemia May Explain Differences in Acidosis-Related Mortality
Source: PLoS One. 2016 Sep 29;11(9):e0163728. doi: 10.1371/journal.pone.0163728 (PMC5042445; doi:10.1371/journal.pone.0163728)
Supplement: S2 Table — (DOCX) [file pone.0163728.s003.docx]

**S2. Table. Parasite Biomass in children with cerebral malaria alone (CM) or cerebral malaria with severe malarial anemia (CM + SMA)**

|  | CM  N=166 | CM + SMA  N=50 | *P^a^* |
| --- | --- | --- | --- |
| Total parasite biomass x 10^8^, median (IQR) | 17,427 (5,747, 37,523) | 33,899 (14,741, 52,028) | 0.003 |
| Circulating parasite biomass x 10^8^, median (IQR) | 643 (170, 3,814) | 222 (103, 1,311) | 0.02 |
| Sequestered parasite biomass x 10^8^, median (IQR) | 13,717 (4,628, 32,886) | 32,155 (14,581, 51,513) | 0.0006 |
|  | CM + SMA Deep Breathing  N=9 | CM + SMA No Deep Breathing  N=41 |  |
| Total parasite biomass x 10^8^, median (IQR) | 34,073 (11,020, 47,232) | 33,725 (15,274, 52,028) | 0.7 |
| Circulating parasite biomass x 10^8^, median (IQR) | 199 (128, 1,997) | 226 (103, 1,250) | 1.0 |
| Sequestered parasite biomass x 10^8^, median (IQR) | 33,033 (10,892, 45,234) | 31,276 (14,645, 51,513) | 0.7 |
|  | CM + SMA Acidosis  N=21 | CM + SMA No Acidosis  N=29 |  |
| Total parasite biomass x 10^8^, median (IQR) | 38,886 (26,186, 61,696) | 27,698 (13,515, 47,298) | 0.2 |
| Circulating parasite biomass x 10^8^, median (IQR) | 190 (55, 1,997) | 226 (136, 1,040) | 0.7 |
| Sequestered parasite biomass x 10^8^, median (IQR) | 38,696 (23,961, 61,327) | 27,621 (12,963, 45,427) | 0.2 |
|  | CM + SMA Died  N=5 | CM + SMA Survived  N=45 |  |
| Total parasite biomass x 10^8^, median (IQR) | 47,232 (35,724, 65,752) | 31,392 (13,626, 48,110) | 0.3 |
| Circulating parasite biomass x 10^8^, median (IQR) | 177 (13, 1,997) | 226 (116, 1,250) | 0.5 |
| Sequestered parasite biomass x 10^8^, median (IQR) | 45,234 (33,658, 65,739) | 29,936 (13,380, 47,109) | 0.3 |

*^a^* P-value for Wilcoxon rank-sum
