# Supplementary material for: Differing Causes of Lactic Acidosis and Deep Breathing in Cerebral Malaria and Severe Malarial Anemia May Explain Differences in Acidosis-Related Mortality
Source: PLoS One. 2016 Sep 29;11(9):e0163728. doi: 10.1371/journal.pone.0163728 (PMC5042445; doi:10.1371/journal.pone.0163728)
Supplement: S3 Table — (DOCX) [file pone.0163728.s004.docx]

**S3 Table. Levels of disease pathogenesis markers in children with cerebral malaria (CM) and severe malaria anemia (SMA) with vs. without deep breathing or lactic acidosis**

|  | Deep breathing  N=10 | No deep breathing  N=46 | P*^a^* |
| --- | --- | --- | --- |
| Platelet Count 10^9^/L, median (IQR) | 38 (29, 137) | 87 (50, 123) | 0.1 |
| PfHRP210^3^ng/ml, median (IQR) | 4,230 (1,632, 6,381) | 3,748 (1,919, 5,887) | 0.9 |
| Hemoglobin, g/dL, mean (SD) | 4.0 (0.8) | 4.1 (0.8) | 0.7 |
| Peripheral blood *P falciparum* density, parasites/μL, median (IQR) | 20,550 (2,060, 226,980) | 21,750 (8,090, 144,520)*^b^* | 1.0 |
|  | Lactic acidosis  N=23 | No lactic acidosis  N=33 |  |
| Platelet Count 10^9^/L, median (IQR) | 61 (34, 123) | 88 (50, 131) | 0.3 |
| PfHRP2 10^3^ng/ml, median (IQR) | 4,394 (2,630, 6,938) | 3,350 (1,733, 5,630) | 0.2 |
| Hemoglobin, g/dL, mean (SD) | 4.0 (0.7) | 4.2 (0.8) | 0.2 |
| Peripheral blood *P falciparum* density, parasites/μL, median (IQR) | 19,840 (1,840, 226,980) | 24,030 (9,920, 161,360)*^b^* | 0.6 |

*^a^* P-value for continuous variables compared by Students’ t-test if normally distributed and Wilcoxon rank-sum if skewed

distribution, and for categorical variables by χ^2^ or Fisher’s exact test where appropriate

*^b^* N differs from total N and is noted in supplementary table 6
