# Supplementary material for: Differing Causes of Lactic Acidosis and Deep Breathing in Cerebral Malaria and Severe Malarial Anemia May Explain Differences in Acidosis-Related Mortality
Source: PLoS One. 2016 Sep 29;11(9):e0163728. doi: 10.1371/journal.pone.0163728 (PMC5042445; doi:10.1371/journal.pone.0163728)
Supplement: S4 Table — (DOCX) [file pone.0163728.s005.docx]

**S4 Table. Demographic, clinical and laboratory findings in children with cerebral malaria (CM) and severe malaria anemia (SMA) who survived vs. died**

| Characteristic or finding | Died  N=6 | Survived  N=50 | P^a^ |
| --- | --- | --- | --- |
| Age in years, mean (SD) | 4.0 (2.9) | 3.2 (1.2) | 0.2 |
| Sex, N (% female) | 4 (66.7) | 19 (38.0) | 0.2 |
| Respiratory distress, N (%) | 4 (66.7) | 20 (40.0) | 0.4 |
| Deep breathing, N (%) | 2 (33.3) | 8 (16.0) | 0.3 |
| Lactic acidosis^b^, N (%) | 4 (66.7) | 19 (38.0) | 0.2 |
| Blood lactate, mmol/L, median (IQR) | 9.5 (3.7, 9.9) | 3.7 (1.9, 7.8) | 0.07 |
| Hemoglobin, g/dL, mean (SD) | 3.7 (0.9) | 4.2 (0.7) | 0.2 |
| O_2_ Saturation, median (IQR) | 98 (95, 98) | 97 (94, 98) | 0.6 |
| O2 Saturation <92%, n (%) | 0 (0) | 8 (16.0) | 0.6 |
| Platelet count, 10^9^/L, median (IQR) | 92 (42, 137) | 76 (39, 121) | 0.7 |
| PfHRP2, 10^3^ng/mL, median (IQR) | 4,583 (2,917, 6,381) | 3,926 (1,735, 5,887) | 0.5 |
| Peripheral blood *P falciparum* density, parasites/μL,,median (IQR) | 10,750 (800, 161,360) | 23,225 (9,050, 158,060)*^c^* | 0.3 |

*^a^* P-value for continuous variables compared by Students’ t-test if normally distributed and Wilcoxon rank-sum if skewed distribution, and for categorical variables by χ^2^ or Fisher’s exact test where appropriate

*^b^* Lactic acidosis defined as blood lactate > 5.0 mmol/liter

*^c^* N differs from total N and is noted in supplementary table 6
