# Supplementary material for: Differing Causes of Lactic Acidosis and Deep Breathing in Cerebral Malaria and Severe Malarial Anemia May Explain Differences in Acidosis-Related Mortality
Source: PLoS One. 2016 Sep 29;11(9):e0163728. doi: 10.1371/journal.pone.0163728 (PMC5042445; doi:10.1371/journal.pone.0163728)
Supplement: S5 Table — (DOCX) [file pone.0163728.s006.docx]

**S5 Table. Mortality in children with cerebral malaria (CM) and severe malaria anemia (SMA) in the presence or absence of deep breathing and lactic acidosis**

|  | CM  N=193 | | | CM + SMA  N=56 | | |
| --- | --- | --- | --- | --- | --- | --- |
| Finding | N with factor | % with factor | Mortality N (%) with factor | N with factor | % with factor | Mortality N (%)^a^ with factor |
| All children | 193 | - | 25 (13.0) | 56 | - | 6 (10.7) |
| DB | 12 | 6.2 | 6 (50.0) | 10 | 17.9 | 2 (20.0) |
| LA | 66 | 34.2 | 13 (19.7) | 23 | 41.1 | 4 (17.4) |
| DB and LA | 12 | 6.2 | 6 (50.0) | 8 | 14.3 | 2 (25.0) |
| DB without LA | 0 | 0 | 0 | 2 | 3.6 | 0 |
| LA without DB | 54 | 28.0 | 7 (13.0) | 15 | 26.8 | 2 (13.3) |
| No DB | 181 | 93.8 | 19 (10.5) | 46 | 82.1 | 4 (8.7) |
| No LA | 127 | 65.8 | 12 (9.5) | 33 | 58.9 | 2 (6.1) |

^a^Mortality did not differ significantly between children with CM and children with CM+ SMA for any factor (all P values >0.05)
