# Supplementary material for: Differing Causes of Lactic Acidosis and Deep Breathing in Cerebral Malaria and Severe Malarial Anemia May Explain Differences in Acidosis-Related Mortality
Source: PLoS One. 2016 Sep 29;11(9):e0163728. doi: 10.1371/journal.pone.0163728 (PMC5042445; doi:10.1371/journal.pone.0163728)
Supplement: S6 Table — (DOCX) [file pone.0163728.s007.docx]

**S6 Table. Numbers tested for subgroup testing in prior tables.**

| Table | Variable, Group, N |
| --- | --- |
| Table 1. | O_2_ saturation and O_2_<92%, SMA, 215; Platelet count, CM, 189, SMA, 214; PfHRP2, SMA, 213; Peripheral blood *P falciparum* density, CM, 189, SMA, 215. |
| Table 3a. | Platelet count, DB, 11, No DB 178, LA, 64, No LA, 125; Peripheral blood *P falciparum* density, DB, 11, No DB, 178, LA, 65, No LA, 124. |
| Table 3b. | Platelet count, No DB, 198, LA, 102, No LA, 112; PfHRP2, DB, 15, No DB, 198, LA, 101, No LA, 112; Peripheral blood *P falciparum* density, No DB, 199, LA, 102. |
| Table 4. | Platelet count, Died, 23, Survived, 166; Peripheral blood *P falciparum* density, Died, 23, Survived, 166. |
| Supplemental table 1. | Platelet count, CM, 189; Peripheral blood *P falciparum* density, CM, 189, CM + SMA, 54. |
| Supplemental table 3. | Peripheral blood *P falciparum* density, CM + SMA no DB 44, CM + SMA no LA, 31. |
| Supplemental table 4. | Peripheral blood *P falciparum* density, Died, 48 |
